# A tailored e-learning program to improve handover in the chain of emergency care: a pre-test post-test study

**DOI:** 10.1186/s13049-015-0113-3

**Published:** 2015-04-16

**Authors:** Remco HA Ebben, Pierre M van Grunsven, Marie Louise Moors, Peter Aldenhoven, Jordan de Vaan, Roger van Hout, Theo van Achterberg, Lilian CM Vloet

**Affiliations:** Research department acute care, HAN University of Applied Sciences, Nijmegen, the Netherlands; Ambulance Service Gelderland Zuid, Veiligheidsregio Gelderland Zuid, Nijmegen, the Netherlands; Emergency Department, Radboud university medical center, Nijmegen, the Netherlands; Centre for Health Services and Nursing Research, KU Leuven, Leuven, Belgium; Scientific Institute for Quality of Healthcare, Radboud university medical center, Nijmegen, the Netherlands; Canisius Wilhelmina Hospital, Nijmegen, the Netherlands

**Keywords:** Guideline adherence [MeSH], Patient handoff [MeSH], Emergency Medical services [MeSH]

## Abstract

**Objective:**

To standardize patient handover in the chain of emergency care a handover guideline was developed. The main guideline recommendation is to use the DeMIST model (Demographics, Mechanism of Injury/illness, Injury/Illness, Signs, Treatment given) to structure pre-hospital notification and handover. To benefit from the new guideline, guideline adherence is necessary. As adherence to guidelines in emergency care settings is variable, there is a need to systematically implement the new guideline. For implementation of the guideline we developed a e-learning program tailored to influencing factors. The aim of the study was to evaluate the effectiveness of this e-learning program to improve emergency care professionals’ adherence to the handover guideline during pre-hospital notification and handover in the chain of emergency medical service (EMS), emergency medical dispatch (EMD), and emergency department (ED).

**Methods:**

A prospective pre-test post-test study was conducted. The intervention was a tailored e-learning program that was offered to ambulance crew and emergency medical dispatchers (n=88). Data on adherence included pre-hospital notifications and handovers and were collected through observations and audiotapes before and after the e-learning program. Data were analyzed using X^2^-tests and t-tests.

**Results:**

In total, 78/88 (88.6%) professionals followed the e-learning program. During pre- and post-test, 146 and 169 handovers were observed respectively. After the e-learning program, no significant difference in the number of handovers with the DeMIST model (77.9% vs. 73.1%, p=.319) and the number of handovers with the correct sequence of the DeMIST model (69.9% vs. 70.5%, p=.159) existed. During the handover, the number of questions by ED staff and interruptions significantly increased from 49.0% to 68.9% and from 15.2% to 52.7% respectively (both p=.000). Most handovers were performed after patient transfer, this did not change after the intervention (p=.167). The number of handovers where information was documented during handover slightly increased from 26.9% to 29.3% (p=.632).

**Conclusions:**

The tailored e-learning program did not improve adherence to a handover guideline in the chain of emergency care. Results show a relatively high baseline adherence rate to usage and correct sequence of the DeMIST model. Improvements in the handover process can be made on the documentation of information during handover, the number of interruptions and questions, and the handover moment.

## Introduction

Patient handover from one health care setting to another includes possible threats to quality and continuity of care [1]. A handover is characterized by the involvement of two or more professionals, the exchange of verbal and/or written information about the patient’s diagnosis, treatment and care, and the transition of patient responsibility [1-5]. The handover from ambulance to emergency department (ED) involves 2-way communication between the ambulance crew and ED-staff [6]. Especially the handover from ambulance to ED seems error prone as there is a high patient turnover, patients present themselves with a wide diversity of clinical conditions, there are acute time constraints, and the ambulance crew has only one opportunity to transfer patient information [7]. Previous studies report a loss of information during handover from ambulance to ED [5,8,9]. Factors which might influence the quality of the handover from ambulance to ED are a lack of active listening skills or inattention of ED-staff, unnecessary repetitions or provision of unnecessary information by ambulance crew, interruptions, workload, working relationships between ambulance crew and ED-staff, uncertainty about the transfer of responsibility, and failure to reach shared understanding [5,6,10-12].

To overcome these problems and barriers, standardization of the handover from ambulance to ED is recommended [5,13]. To facilitate standardization, structured models for ambulance to ED handover have been developed: (De)MIST (Demographics, Mechanism of Injury/illness, Injury or Illness found or suspected, Signs, Treatment given), AMPLE (Allergies, Medications, Past illnesses, Last meal, Events), ASHICE (Age, Sex, History, Injuries, Condition, Expected time of arrival), IMIST-AMBO (Identification of the patient, Mechanism/medical complaint, Injuries, Signs, Treatment and treatment respons/trend, Allergies, Medications, Background and Other), SOAP (Subjective information, Objective information, Assessment, Plan) and BAUM (‘Bestand’ (inventory), ‘Anamnese’ (medical history), ‘Klinische Untersuchungsergebnisse’ (clinical findings), ‘Massnahmen’ (actions)) [5,13-15].

To standardize handover practice from ambulance to ED in the Netherlands, an evidence-based guideline has been developed. The key-recommendation of the guideline is to use the DeMIST-model to structure prehospital notification and handover in the chain of ambulance, emergency medical dispatch (EMD) and ED. Due to a lack of evidence about effectiveness and applicability of handovers models, the choice for the DeMIST model was based on the fact that the MIST model was already in use. Other recommendations of the handover guideline are (a) that the pre-hospital professional who is responsible for the patient, provides a handover to the ED-professional who will be responsible for the patient, (b) that a handover takes place before patient transfer, and (c) that the ambulance crew verifies if the handover was clear.

To assist implementation of the newly developed guideline, a tailored e-learning program was developed to serve as educational intervention. The e-learning program was tailored to influencing factors that were identified beforehand in the local chain of emergency care. Previous studies show that e-learning can be effective to teach emergency physicians and nurses to recognize child abuse, to administer metoclopramide and to improve triage skills [16-18]. The effect of e-leaning on handover has not been studied.

Therefore, the aim of the study was to evaluate the effectiveness of an e-learning program to improve adherence to the handover guideline, hereby structuring pre-hospital notification and handover in the chain of ambulance-EMD-ED. We hypothesized that the e-learning was effective to improve handover on the two key-guideline recommendations (Table 1), being 1) to use the DeMIST model and 2) to use the DeMIST model in the correct sequence.

**Table 1:** **Outcomes**

**Primary outcomes (N1 + N2 + O1)**	**Origin**	**Scoring options**
Handover model used	Key-guideline recommendation	DeMIST/Other
Correct sequence of DeMIST model	Key-guideline recommendation	Yes/no/Specification of sequence if incorrect
**Secondary outcomes (O1)**		
Sender of the handover	Guideline recommendation	Ambulance nurse/ambulance driver
Composition of the receiving team	Guideline recommendation	ED-physician/ED-nurse/team
Recognizability of the receiver	Guideline recommendation	Optic/verbal/none
Handover moment	Guideline recommendation	Before/during/after patient transfer
Verification if handover was clear	Guideline recommendation	Yes/no
Documentation of handover	Literature	Whiteboard/DeMIST-form/patient file/different
No. of clarifying questions asked by receiver	Literature	Actual number
No. of repetitions from sender	Literature	Actual number
No. of interruptions other than questions or repetitions	Literature	Actual number

## Methods

### Design

The study had a prospective pre-test post-test design.

### Setting

The study setting was located in the chain of emergency care of Nijmegen, the Netherlands. The chain of emergency care consists of the regional ambulance service (EMS), the emergency medical dispatch centre (EMD), and the emergency department (ED) of the Radboud university medical centre. In 2013, the EMD in this region managed 66.316 ambulance calls. Ambulances are staffed with one driver and one registered ambulance nurse, specialized EMD-nurses staff the EMD. Registered nurses become qualified as an ambulance nurse or EMD-nurse after following a specific national training course. Ambulance nurses work autonomously and are allowed to administer medical treatment based on their national protocol, without direct consultation of an EMS physician. The ED of the Radboudumc is a level 1 traumacenter, meaning the ED is delivering 24/7 trauma care for all types of patients. The ED had 21.672 admissions in 2012 and is staffed with emergency nurses and emergency physicians. Additional medical teams (trauma surgeon, intensivist) can be activated.

### Handover process

**Figure 1:**
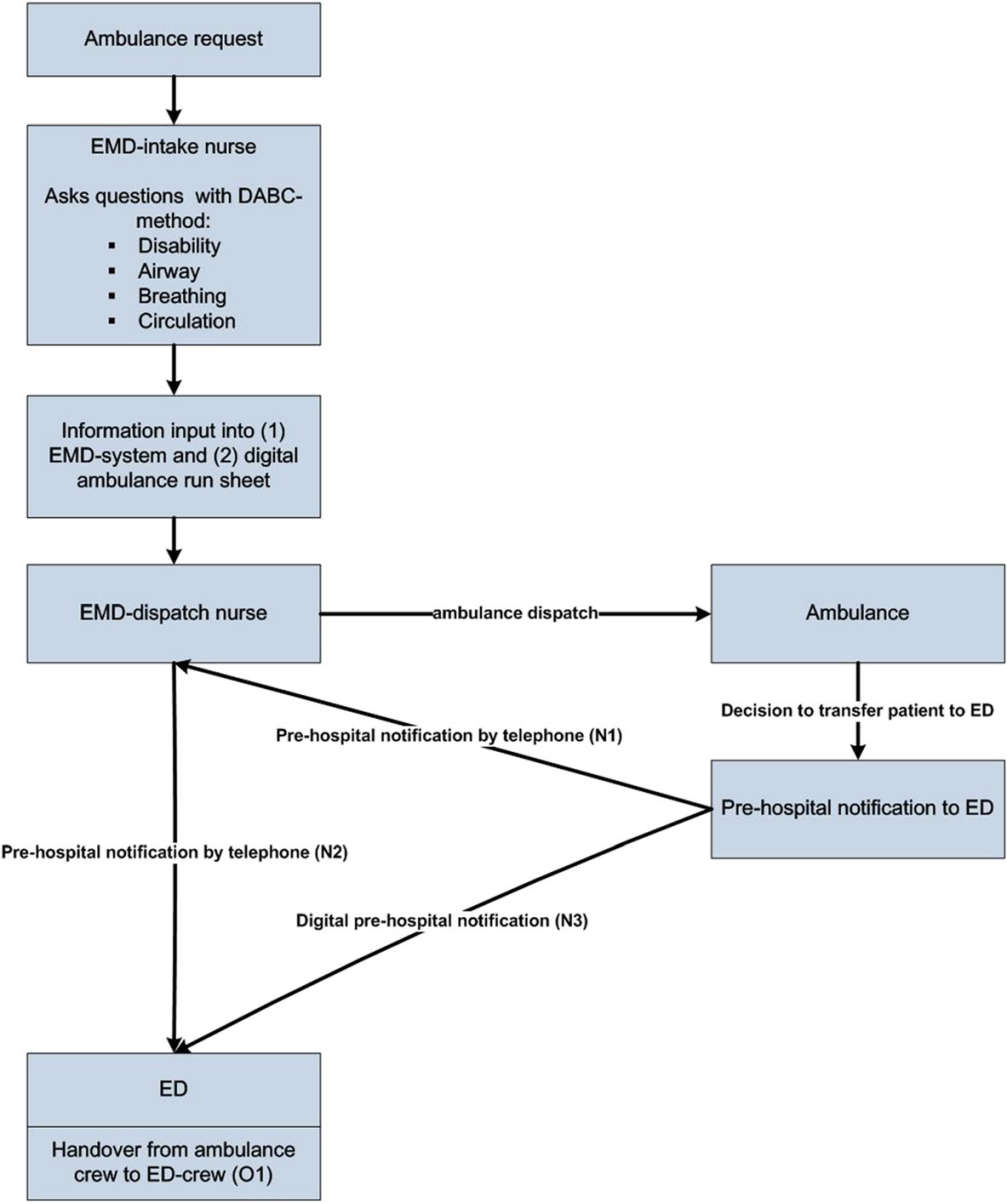
Patients transfer and handover.

The transfer process of the patient and patient information in the chain of emergency care is displayed in Figure 1. A request for an ambulance can be made by a lay person calling the national emergency number, or by another healthcare professional (e.g. general practitioner). The request is handled by the EMD-intake nurse, who interrogates the caller and triages the problem. During the intake, information is stored into (1) the EMD-system and (2) the digital ambulance run sheet. On the basis of this information and guided by dispatch-protocols, the EMD-dispatch nurse decides about dispatching an ambulance. The ambulance can be dispatched with urgency level A1 (arrival <15 minutes), A2 (arrival <30 minutes) or B (planned). After on-scene diagnosis and treatment, the ambulance nurse may decide to transfer the patient to the ED. The ambulance crew provides a notification (N1) to the EMD-dispatch nurse by telephone, then the EMD-dispatch nurse calls the ED and notifies the ED (N2). Both calls are logged into the EMD-system. At the same time, the ambulance sends a digital notification (N3) to the ED. The digital notification is a short version of the ambulance run sheet with DeMIST data. When the ambulance arrives at the ED, the handover of the patient, the patient information, and the patients’ personal belongings from ambulance to ED takes place (O1).

### Outcomes

The degree of adherence to the key-recommendation to use the DeMIST-model in the correct sequence to structure pre-hospital notification and handover from ambulance to ED was the primary outcome of this study for N1, N2 and O1 (Table 1). For handover (O1), secondary outcomes based on guideline recommendations were the professional providing a handover (sender), the composition of the receiving team, recognizability of the receiver, handover moment, and verification if the handover was clear. The choice for ‘composition of the receiving team’ was also based on the idea that if the receiving team is complete at the start of a handover, this reduces the risk of loss of information due to multiple handovers (‘Chinese whisper’) [5]. Additional secondary outcomes based on literature were documentation of information from the handover, the number of repetitions, number of questions, and the number of interruptions other than repetitions and questions.

### Pre-test

The pre-test phase consisted of two periods of 4 weeks between April 22nd and August 9th 2013. Handovers from ambulance to ED for all types of ambulance runs and all types of patients were included. Handovers of patients with a possible or confirmed MRSA-contamination were excluded, as these handovers took place in separate rooms with infection precautions. Data were collected in two steps. First, all handovers from ambulance to ED were observed using a structured data collection form based on primary and secondary outcomes and scoring options (Table 1). Secondly, to collect data for the prehospital notification (N1 + N2) from ambulance to ED we used audiotapes. Corresponding audiotapes for a handover were identified at the EMD using an unique ambulance run identifier. All data were collected by trained 4^th^ year students from the bachelor of nursing or bachelor of health studies.

### Intervention

We used Grol's model for effective implementation as study framework (Figure 1) [19,20]. The model provides a stepwise approach for improving clinical practice and starts with the identification of research findings or guidelines that have to be implemented (step 1). Steps 2 and 3 include a description of (change) targets and an analysis of the target group, current practice and setting. On the basis of this analysis, implementation strategies can be selected or developed (step 4), followed by the execution and evaluation of an implementation plan (steps 5 and 6). For step 1 in this study, the aim was to implement the handover guideline. The analysis of the target group (step 2 and 3) was undertaken composing a multi-disciplinary steering group and conducting a focus group interview. The multi-disciplinary steering group included opinion leaders from the chain of emergency-care: 1 EMD nurse, 1 ambulance nurse, 1 ambulance care medical manager, 1 emergency physician and 1 emergency nurse. The role of the steering group was to provide input in the study design and to create support for the study in the local chain of emergency care. The aim of the focus group interview was to identify handover problems in the local chain of emergency care. The focus group was organized in April 2013, participants were 2 emergency physicians, 2 emergency nurses, 2 ambulance nurses and 1 EMD-nurse. The focus group interview was audiotaped, and transcribed verbatim. Two researchers identified the problems on the basis of the transcription, and for each problem a key-determinant was added (Table 2). To enhance trustworthiness a member check was performed [21], therefore identified problems and determinants were sent to the participants of the focus group interview. Furthermore, problems which arose from the pre-test were also addressed.

**Table 2:** **Handover problems identified in the chain of emergency care**

**Focus group interview**	**Determinants**
● Non-usage of the DeMIST model	Knowledge, skills and motivation on:
Incorrect sequence of the DeMIST model	● How to use the DeMIST for all types of patients (trauma and non-trauma)
● Difficulties with applying the DeMIST model to trauma and non-trauma patients	
	● The correct sequence of DeMIST
● Handover of subjective information/interpretation of information (“patient is stable) instead of objective parameters	● Usage of objective information
	● The timing of the handover
● Ambulance crew has the impression that the digital notification is only used for retrieval of patient information and not for monitoring the patient	● The documentation of handover
	● The advantages of using the DeMIST model in the chain of emergency care
● Unclear for ambulance crew who is the receiver of the handover at the ED	
**Pre-test**	
● 77.9% of the handovers were structured with the DeMIST model	
● 69.9% of the DeMIST handovers had the correct sequence	
● 73.1% of the handovers took place after patient transfer	
● 49% of the handovers were interrupted by questions from ED-staff	
● 26.9% of the handovers were documented	

On the basis of identified problems and determinants the steering group chose to use a tailored online e-learning program as intervention. Literature suggests tailoring interventions to identified problems to increase effectiveness, although the effectiveness of tailoring has not been proven irrefutable yet [22]. Reasons to choose e-learning were (a) the fact that all determinants could be addressed, (b) the flexibility, availability and accessibility of using e-learning which suits the emergency care context [23], and (c) the fact the target group was familiar with e-learning in their training programs. The e-learning program was specifically designed for EMD-nurses, ambulance nurses and drivers, emergency nurses and physicians, on the basis of (1) the handover guideline, (2) literature, (3) expert opinion, and (4) identified problems. The e-learning program yielded five components aimed at (1) knowledge about the DeMIST model and handover process, (2) skills how to use the DeMIST model to provide a proper handover, and (3) motivation to use the DeMIST model in the total chain of emergency care (Table 3).

**Table 3:** **Components and content of the e-learning program**

**Component**	**Aim**	**Content**
Introduction	Explanation on the usage of the e-learning program and the learning goals to the participant	Learning goals
		● The professional knows the elements of a proper DeMIST handover
		● The professionals knows why it is important to use the DeMIST model for handover
		● The professional knows the role of the emergency medical dispatcher, ambulance driver, ambulance nurse, emergency nurse and emergency physician during handover
		● The professional performs a DeMIST handover for trauma and non-trauma patients
Theory	Provision of theory on DeMIST and its usage to the participant	Theory on DeMIST
		● De: full name, date of birth/age and sex of the patient
		● M: trauma or non-trauma
		● I: injuries found or suspected/complaints
		● S: Airway, Breathing (frequency, SpO_2_), Circulation (heart rate, blood pressure), and Disability (EMV-score, pupil reaction, pain, blood glucose)
		● T: working diagnosis, treatment given, effect of the treatment
		When to use DeMIST
		How to use DeMIST
		Supply of objective information
		When to provide a handover
		Verification if handover was clear
		Who provides a handover to whom (professionals’ role)
		Advantages of using the DeMIST in the total chain of emergency care
Knowledge test	Summative test whether the participant has sufficient knowledge, insight and basic skills about a DeMIST handover	8 random questions out of 22 on knowledge, insight and application of theory
Simulation test	Summative test whether the participant can integrate knowledge and skills to provide a DeMIST handover	2 high fidelity simulation scenarios, randomly picked from 7 possible scenarios:
		1. Female (75 yrs), low energetic trauma (pedestrian-car)
		2. Male (45 yrs), high energetic trauma (tree-car)
		3. Female (28 yrs), hypovolemic shock (fluxus post partum)
		4. Male (30 yrs), fever, hypotension altered consciousness (septicaemia)
		5. Female (55 yrs), resuscitation
		6. Male (68 yrs), resuscitation
		7. Female (70 yrs), stroke
Evaluation	Feedback on knowledge and simulation test to the participant	Achievement on learning goals with feedback

During the knowledge test, 8 random questions were presented. The caesura for the knowledge test was 87.5% (1 wrong answer). The e-learning program included 7 simulation scenarios on trauma (2x), resuscitation (2x), septicaemia (1x), fluxus post-partum (1x), and stroke (1x). All scenarios were designed with regard to the 3 diagnoses with the highest national incidence in emergency care: cardiology, trauma and internal medicine. The scenarios could be simulated from EMD, EMS or ED perspective that the professional could choose. This choice was added to the e-learning program to emphasize the chain of emergency care. Professionals could exercise simulations before entering the simulation test. During the simulation test the professionals had to simulate two scenarios (caesura 90%). The construction of the simulation test included that the result of the first scenario cumulated to the result of the second scenario, so if the professional failed the first simulation, zero faults were allowed during the second simulation. The rationale was that professionals provided a good handover the first time, as in real practice the ambulance crew has only one opportunity to provide a handover. Both the knowledge and simulation tests provided feedback to the professionals. The draft version of the e-learning program was tested on content and usability by representatives (n = 6) from the targetgroup. There was no maximum time restriction for completion of the entire e-learning program.

### Intervention phase

All possible professionals from the EMD (n = 15) and EMS (n = 73) who could be involved in a handover during the study period were invited to follow the e-learning program on October 3rd 2013. The e-learning program was accompanied by an email in which the purpose was explained, and in which the professionals were motivated by their managers and educational coordinators. Also, information on the EMD and EMS intranet was published and professionals were motivated to follow the e-learning program by members of the steering group. The professionals could start the e-learning program any time on any computer they wanted, until November 16th 2013. During this period, each professional received 2 digital reminders. To stimulate the professionals to follow the e-learning program, the program was accredited with official registration points for EMD and ambulance nurses, and ambulance drivers.

### Post-test

The post-test phase lasted from November 11th until December 8th 2013. To collect data, the same methods were used as in the pre-test.

### Data analysis

As this study is the first intervention study on handover, the number of observed handovers was based on feasibility and we did not perform a formal power analysis. To have an estimation, the minimum number of handovers required was determined by a power analysis using G*Power 3 [24]. Hereby, we set the α-level at .05 and the power level at .8. Based on these settings, we needed 143 handovers. Data were entered in SPSS and analysed using descriptive techniques. To compare the pre-test data with the post-test data, X^2^-tests and t-tests were performed. For all tests, statistical significance was set at P-value less than 0.05. To enhance validity and reliability, all handovers and audiotapes were observed/listened by two independent observers who discussed differences until consensus was reached. Inter-rater reliability was computed for a random sample of 10% (n = 17) of the observations in the post-test and was 91.9%.

### Ethical considerations

On the basis of the study protocol, the Committee on Research Involving Human Subjects region Arnhem/Nijmegen waived the need for ethical approval (registration number 2013/046).

## Results

### Intervention

In total, 78/88 (88.6%) professionals followed the e-learning program, of which 70/78 (89.7%) certified for the knowledge test, and 41/70 (52.6%) certified for the simulation test also. The professionals spent an average median time on the e-learning program of 75 minutes (Table 4).

**Table 4:** **Characteristics of the e-learning program**

**Variable**			
**All professionals (n = 88)**			**n (%)**
Started the e-learning program			78 (88.6)
**Certification status of starters (n = 78)**			**n (%)**
Knowledge test alone			29 (37.2)
Knowledge test + simulation test			41 (52.6)
Started but no certification			8 (10.3)
**Average time spent of starters (n = 78)**	**25th percentile**	**Median**	**75th percentile**
Time spent on theory (in minutes)	5	11	18
Time spent on knowledge test (in minutes)	15	30	46
Time spent on simulation test (in minutes)	14	34	115
*Total time spent (in minutes)*	*47*	*75*	*181*

### Handover from ambulance to ED

All observed handovers during the pre-test (n = 145) were included. From the observed post-test handovers (n = 169), two handovers were excluded as these were provided by EMS-students who did not work at the EMS during the intervention period, leaving a total of 167 handovers. There were no significant differences between both study periods regarding patient gender, medical specialty or urgency.

Regarding the primary outcome, no significant difference in the number of handovers that were structured with the DeMIST model between the pre-test (77.9%) and the post-test (73.1%) existed (Table 5). In the pre-test, 69.9% of the DeMIST handovers used a correct sequence, in the post-test this was 70.5%. When professionals deviated from the correct sequence during both the pre- and post-test, the most common deviation was to mix or switch the ‘S’ and ‘T’. There was no association between medical specialty (trauma/non-trauma) and the correct sequence (X^2^ = .36, p = .872).

**Table 5:** **Handover from ambulance to ED (O1)**

**Variable**	**Pre-test (n = 145)**	**Post-test (n = 167)**	**p-value X2-test**
	**n (%)**	**n (%)**	
**Characteristics**			
**Patient gender**			.797
Male	75 (51.7)	83 (49.7)	
Female	70 (48.3)	73 (43.7)	
Missing*		10 (6.0)	
**Medical specialty**			.106
Trauma	32 (22.1)	25 (15.0)	
Non-trauma	113 (77.9)	142 (85.0)	
**Urgency**			
A1 (within 15 minutes)	49 (33.8)	44 (26.3)	.152
A2 (within 30 minutes)	81 (55.9)	93 (55.7)	.975
B (low urgency/planned)	15 (10.3)	30 (18.0)	.056
**Primary outcomes**			
**Handover model used**			
DeMIST	113 (77.9)	122 (73.1)	.319
ABCD	0 (0.0)	2 (1.2)	-
AMPLE	0 (0.0)	1 (0.6)	-
No method/not recognizable	32 (22.1)	42 (25.1)	.523
**Correct sequence of the DeMIST**			
Yes	79 (69.9)	86 (70.5)	.159
No	34 (30.1)	24 (19.7)	
No sequence recognizable within DeMIST	-	12 (9.8)	
**Secondary outcomes**			
**Receiving team composition at start handover**			
Physician and nurse	62 (42.8)	68 (40.7)	.715
Physician later than start	64 (44.1)	90 (53.9)	.035**
Nurse later than start	18 (12.4)	9 (5.4)	.055
Physician and nurse too late	1 (0.7)	-	-
**Receivers recognizability**			
Optic	33 (22.8)	39 (23.4)	.901
Verbal	29 (20.0)	49 (29.3)	.057
Not recognizable	83 (57,2)	79 (47.3)	.080
**Handover given by**			
Ambulance nurse	143 (98.6)	157 (94.0)	.035**
Ambulance nurse + ambulance driver	2 (1.4)	10 (6.0)	
**Receiver handover**			
ED physician	19 (13.1)	26 (15.6)	.553
ED nurse	64 (44.1)	89 (53.3)	.120
ED team(minimum: ED nurse + ED physician)	61 (42.1)	52 (31.1)	.040**
Missing	1 (0.7)	-	-
**Handover moment**			
Before patient transfer	28 (19.3)	40 (24.0)	.322
During patient transfer	3 (2.1)	9 (5.4)	.128
After patient transfer	106 (73.1)	110 (65.9)	.167
Different (patient is to toilet or is in different room)	8 (5.5)	8 (4.8)	.772
**Number of handovers with repetitions**	17 (11.8)	21 (12.6)	.819
**Number of handovers with questions**	71 (49.0)	115 (68.9)	.000**
**Number of handovers with interruptions**	22 (15.2)	88 (52.7)	.000**
**Number of handover were verification was asked**	22 (15.2)	19 (11.4)	.322
**Handover documented**	39 (26.9)	49 (29.3)	.632
Whiteboard	0 (0.0)	2 (4.1)	-
DeMIST form	1 (2.6)	3 (6.1)	-
Patient file	0 (0.0)	1 (2.0)	-
Different***	38 (97.4)	43 (87.8)	.095

*The gender was not registered for 10 patients, this could not be retrieved.

**Significant difference.

***Glove, napkin, sheet, paper.

The composition of the receiving team differed after the intervention as less handovers started with presence of an emergency physician. There was no difference in how the receivers made themselves recognizable. During the post-test, significantly more handovers in which the ambulance driver was involved took place. Between pre- and post-test, there were no significant differences between the moment of the handovers, although the highest number of handovers take place after patient transfer. After the intervention, the number of handover with questions (p = .000) and interruptions (p = .000) significantly increased, the number of handovers with repetitions did not differ. The percentage of handover with a verification did not significantly decrease.

### Pre-hospital notification

During the pre-hospital notification from ambulance to EMD (N1), no significant difference in the number of handovers that were structured with the DeMIST model between the pre-test (72.9%) and post-test (80.7%) existed (Table 6). In the pre-test, 66.7% of the DeMIST handovers used a correct sequence, in the post-test this was 56.5%. During the pre-hospital notification from EMD to ED (N2), no significant difference in how many handovers were structured with the DeMIST model between the pre-test (83.3%) and post-test (86.5%) existed. In the pre-test, 84.0% of the DeMIST handovers used a correct sequence, in the post-test this was 73.3%.

**Table 6:** **Prehospital notification (N1 + N2)**

	**Pre-test (n = 145)**	**Post-test (n = 167)**	**p-value X** ^**2**^ **-test**
	**n (%)**	**n (%)**	
**EMS to EMD by telephone (N1)**			
**Notification given**	70 (48.3)	57 (34.1)	
**Handover model used**			
DeMIST	51 (72.9)	46 (80.7)	.147
ABCD	1 (1.4)	-	-
AMPLE	1 (1.4)	-	-
No method/not recognizable	17 (24.3)	11 (19.3)	.500
**Correct sequence of the DeMIST**			
Yes	34 (66.7)	26 (56.5)	.304
No	17 (33.3)	20 (43.5)	
**EMS to ED by telephone (N2)**			
**Notification given**	60 (41.4)	52 (31.1)	
**Handover model used**			
DeMIST	50 (83.3)	45 (86.5)	.149
ABCD	1 (1.7)	-	-
AMPLE	-	-	-
No method/not recognizable	9 (15.0)	7 (13.5)	.817
**Correct sequence of the DeMIST**			
Yes	42 (84.0)	33 (73.3)	.203
No	8 (16.0)	12 (26.7)	

## Discussion

This study evaluated the effectiveness of a tailored e-learning program to improve adherence to a handover guideline in the chain of emergency care. A total of 314 handovers from ambulance to ED were observed and results show no significant differences regarding the usage and correct sequence of the DeMIST model between the pre-test and post-test.

Results from both the pre-test and post-test phase show adherence rates to the DeMIST model ranging from 77.9%-73.1%, and adherence rates for correct sequence ranging from 69.9%-70.5%. To our knowledge, no studies investigated adherence to an ambulance to ED handover model in real practice, only one study assessed adherence the ISBAR handover model in a simulated setting, reporting an improvement in correct sequence from 0%-46% after a high-fidelity simulation intervention [25]. Compared to other guideline adherence rates in the prehospital and ED setting, adherence in our study is relatively high [26]. Nevertheless, the results also indicate room for improvement as in 22.1%-25.1% of the handovers no model was recognizable. This might incorporate the risk for loss or deformation of essential information. Possibly, this result indicates that professionals might perceive that the DeMIST model does not fit entirely for all patients handed over from ambulance to ED. For instance, one often heard counter argument for the (De)MIST is that it might be less applicable to non-trauma or non-critical patients [13], however our results show no association between trauma or non-trauma and the correct sequence of the DeMIST. On the other hand, one might argue that there are no valid reasons to deviate from a handover guideline, in contrast to diagnostic or therapeutic guidelines and protocols where deviations on the basis of patient conditions or preferences can be justified.

The e-learning program was not effective in improving and thereby implementing the new guideline, this can be explained by several reasons. The first reason might be the relatively high baseline adherence rates. These high rates can be caused by the (De)MIST integration in basic emergency care education in the Netherlands. Another reason might be the sole use of the e-learning program as the sole use of an educational intervention might not be effective [20]. However, emergency care research shows moderate to good effects of the sole use of e-learning [16-18]. Furthermore, our results might also urge the use of blended-learning were e-learning is combined with face-to-face educational meetings [23]. Despite these results, the effectiveness of e-learning should be further investigated as it is widely used to educate and train emergency care professionals [23].

A third reason might be that only the handover senders (EMD and ambulance professionals) were trained. During the study period it was not possible to train the ED-staff because they already were in training for the implementation of a digital patient file. A previous study showed that information retention by ED-staff decreased from 56.6% to 49.2% if the handover model is implemented in the ambulance setting only [7]. This stresses the need to implement a handover model in the chain of emergency care.

A fourth reason is the fact that 88.6% of the professionals started the e-learning program and that 52.6% of the starters certified for the whole program. This means that the intervention did not fully reach all intended professionals. The variation around the median time spent on the simulation test reflects the struggle professionals had with certifying for the simulation test, which was due to the accumulation of the result of scenario 1 with scenario 2. This accumulation resulted in a significant amount of the professionals who had to try several times before certifying for the simulation test which caused the high variation, and in 37.2% of the professionals who only certified for the knowledge test.

Despite relatively high adherence rates for the key-guideline recommendation, our results indicate several areas of improvement for handover from ambulance to ED. Firstly, in 26.9%-29.2% of the handovers transferred information was documented by ED-staff during handover. Most information was written down on gloves, napkins, pieces of paper or sheets, which carries the risk that this information is not integrated in medical records. In our study medical records were not checked for documented information after handover, but previous studies indicate suboptimal documentation of transferred information [9,27].

Secondly, the number of handovers in which verification was asked by ambulance staff, dropped from 15.2% to 11.4%. A previous simulation study also showed low rates of verification, although these rates increased after simulation exercises [25]. Verification of a handover indicates the end of the handover and might prevent interruptions of the handover due to questions asked by ED-staff.

Thirdly, most handovers took place after patient transfer in the ED. Handing over a patient during or after transfer, incorporates the risk that ED-staff already starts diagnostic or therapeutic actions that might distract ED-staff from the handover.

Fourthly, in 44.1%-53.9% the complete team was not present at the start of the handover. A previous study reported physician absence at 88% of the handovers [28]. Our results might be caused by lower-acuity patients for whom it is less urgent to be seen by a physician. Another explanation might be that there is no pre-hospital notification given by the ambulance crew to ED, as with 41.4% of the handovers in the pre-test and 31.1% of the handovers in the post-test a verbal notification was given. Another reason can be that the digital pre-hospital notification (N3) arrives too late at the ED sometimes, and the handover already took place.

Fifthly, the number of handovers with questions from ED-staff and interruptions significantly increased after the intervention. This might be caused by the fact that only the senders of the handover were trained, and that the receiving ED-staff had to get used to the structure. Most of the questions were related to the vital signs (‘S’) and treatment (‘T’). The treatment given is marked by emergency nurses and physicians as an essential element of the handover [6], this might explain the questions. In this study we were not able to mark the questions and repetitions as contributing to the handover or disturbing the handover. Repetitions and questions might contribute to the handover as they can clarify treatment, and lead to hearing specific aspects of the handover again [6]. On the other hand, repetitions and questions might disturb a handover as they might reflect a lack of listening skills or inattention of ED-staff, and ambulance staff gets frustrated if they have to repeat themselves, or as their findings are questioned [6,13]. Most of the interruptions were related to the patient or patients' next of kin. There were relevant interruptions (changing or adding information), and non-relevant interruptions which that were caused by the patient talking (with next of kin or the ambulance driver), phones ringing, and the arrival of other professionals.

### Strengths and weaknesses

Obviously, the absence of a control group might be a threat to the external validity. Another threat to the validity of this study is the Hawthorne effect: the ambulance crew and ED personnel could see the observers when they were present at the ED. Furthermore, not all staff participated in the full intervention, which could explain limited effects. Also, it is possible there are other determinants that influence handover which were not integrated in the e-learning program, making the e-learning program less powerful. Inter-rater reliability between 2 observers was calculated for 10% of the observations, showing a satisfying 91.9% agreement. To increase reliability between pre- and post-test, observers of the post-test were trained by observers from the pre-test, but despite this effort slight differences in observations between pre- and post-test might have occurred. Finally, statistical significance may have occurred due to multiple X^2^-testing, although in the light of the low number of significant tests this did not seem a major issue.

### Future research

Future research should focus on the applicability of different handover models to structure the handover in the chain of emergency care. Which models are applicable for which settings and patients groups? Also, the additional effect of training the receivers of the handover (ED-staff) should be investigated. Furthermore, the use of multiple strategies or blended learning should be examined for their effectiveness to improve handover practice. Finally, the user satisfaction of e-learning to implement a (handover) guideline can be evaluated.

## Conclusion

This pre-test post-test study found no effect of a tailored e-learning program on adherence to a handover guideline. The results suggest that e-learning alone does not improve adherence. Despite the relatively high baseline adherence, our results indicate room for improvement in the handover process, with regard to documentation of information during the handover, the handover moment, and the completeness of the receiving team at the start of the handover.
